# Image Decomposition Algorithm for Dual-Energy Computed Tomography via Fully Convolutional Network

**DOI:** 10.1155/2018/2527516

**Published:** 2018-09-05

**Authors:** Yifu Xu, Bin Yan, Jingfang Zhang, Jian Chen, Lei Zeng, Linyuang Wang

**Affiliations:** ^1^National Digital Switching System Engineering & Technological R&D Centre, Zhengzhou 450002, China; ^2^153 Central Hospital of Henan Province, Zhengzhou 450002, China

## Abstract

**Background:**

Dual-energy computed tomography (DECT) has been widely used due to improved substances identification from additional spectral information. The quality of material-specific image produced by DECT attaches great importance to the elaborated design of the basis material decomposition method.

**Objective:**

The aim of this work is to develop and validate a data-driven algorithm for the image-based decomposition problem.

**Methods:**

A deep neural net, consisting of a fully convolutional net (FCN) and a fully connected net, is proposed to solve the material decomposition problem. The former net extracts the feature representation of input reconstructed images, and the latter net calculates the decomposed basic material coefficients from the joint feature vector. The whole model was trained and tested using a modified clinical dataset.

**Results:**

The proposed FCN delivers image with about 60% smaller bias and 70% lower standard deviation than the competing algorithms, suggesting its better material separation capability. Moreover, FCN still yields excellent performance in case of photon noise.

**Conclusions:**

Our deep cascaded network features high decomposition accuracies and noise robust property. The experimental results have shown the strong function fitting ability of the deep neural network. Deep learning paradigm could be a promising way to solve the nonlinear problem in DECT.

## 1. Introduction

Conventional single-energy X-ray technique provides information about the examined object which is not sufficient to characterize it precisely. Dual-energy computed tomography (DECT) provides additional information by using two different energy spectra to scan the object, which has been presented as a valid alternative to conventional single-energy X-ray imaging. In recent years, the adoption of DECT has gained increased attention in public security [1] and medical field [2, 3]. The advantage of DECT is the ability for material characterization and differentiation [4]. This decomposition of mixture into two basic materials depends on the principle that the attenuation coefficient is material and energy dependent. Thus, measurements at two distinct energies should permit the separation of the attenuation into its basic components.

The quality of material-specific image produced by DECT attaches great importance to the elaborated design of the basis material decomposition method. The existing decomposition methods can be divided into two main categories: projection-based [5–7] and image-based [8–10]. Projection-based methods pass the projection data through a decomposition function, followed by image reconstruction such as filtered backprojection (FBP). It commonly provides better accuracy and reconstructed image with reduced beam-hardening artifacts in comparison with image-based methods. However, projection-based methods need matched projection datasets. This means that physically the same lines need to be measured for each spectrum, which is usually not the case in today's CT scanners. Image-based methods use linear combinations of reconstructed images to get an image that contains material-selective DECT information. It is an approximative technique, and the resulting images are less quantitative than with projection-based methods. But image-based methods can handle mismatched projection datasets and are applicable to the decomposition of three or more constituent materials, which is more expedient in practice. Thus, they have been employed more frequently in modern DECT implementations.

The material decomposition problem in image domain can be described by the following equation:

(1) $$\begin{array}{c} \left(\begin{array}{c} \mu_{\text{H}}\\ \mu_{\text{L}} \end{array}\right)=\left(\begin{array}{cc} {\bar{\mu }}_{1\text{H}} & {\bar{\mu }}_{2\text{H}}\\ {\bar{\mu }}_{1\text{L}} & {\bar{\mu }}_{2\text{L}} \end{array}\right)\left(\begin{array}{c} x_{1}\\ x_{2} \end{array}\right), \end{array}$$

where *μ*_H_ and *μ*_L_ are the pixels in reconstructed images from low- and high-energy projections, respectively, and *x*_1_ and *x*_2_ are the corresponding points in decomposed basic materials images. The subscripts 1 and 2 indicate two specific materials. ${\bar{\mu }}_{1\text{L}/\text{H}}$ and ${\bar{\mu }}_{2\text{L}/\text{H}}$ are the average attenuation coefficients of the two basic materials under low/high-energy spectra. These attenuation coefficients are usually obtained by manually selecting two uniform regions of interest (ROIs) on the CT images that contain the basic materials [9, 11, 12]. Direct material decomposition via matrix inversion is a way of calculating the points *x*_1_ and *x*_2_ in the decomposed image, which is written as follows:

(2) $$\begin{array}{c} \left(\begin{array}{c} x_{1}\\ x_{2} \end{array}\right)=\frac{1}{{\bar{\mu }}_{1\text{H}}{\bar{\mu }}_{2\text{L}}-{\bar{\mu }}_{2\text{H}}{\bar{\mu }}_{1\text{L}}}\left(\begin{array}{cc} {\bar{\mu }}_{2\text{L}} & -{\bar{\mu }}_{2\text{H}}\\ -{\bar{\mu }}_{1\text{L}} & {\bar{\mu }}_{1\text{H}} \end{array}\right)\left(\begin{array}{c} \mu_{\text{H}}\\ \mu_{\text{L}} \end{array}\right). \end{array}$$

Equation (2) can be easily solved as long as the value of $\Delta ={\bar{\mu }}_{1\text{H}}{\bar{\mu }}_{2\text{L}}-{\bar{\mu }}_{2\text{H}}{\bar{\mu }}_{1\text{L}}$ is not null. However, values of the two terms in Δ do not differ significantly from each other. Therefore, the decomposition result is very sensitive to the noise in the input reconstructed images. Various methods have been proposed to solve this noise suppression problem. Precorrection [13, 14] methods reconstruct two water-precorrected images, followed by a linear combination, to yield images that are free from cupping artifacts usually in water-equivalent materials. The noise reduction techniques after image decomposition include Kalender's correlated noise reduction (KCNR) [15, 16], noise forcing (NOF) [17], and noise clipping (NOC) [18], whose most fundamental strategy is the application of a smoothing filter. Recent advanced iterative methods [9, 10] consider the statistical properties of the decomposition process, producing high-quality edge-preserving images. These methods have shown great success on the decomposition problem. Their well performances rely on the well-handcrafted design of the algorithm.

In recent years, deep learning techniques, which use neural networks having a deep structure with three or more layers, have attracted widespread attention, mainly by outperforming alternative machine learning methods in numerous important applications. The current most popular deep model is the convolutional neural network (CNN) which has emerged as a powerful class of models for image classification [19, 20] and object detection [21]. In the field of computed tomography, some of the recent studies have already attempted to use deep neural networks to solve the problems such as low-dose image denoising [22] and artifact reduction [23]. Wang [24] provides an analytical and global perspective to the combination of tomographic imaging and deep learning. For the material decomposition problem in DECT, several neural network-based methods have also been proposed, but they all decompose the material in the projection domain [7, 25, 26].

Inspired by the recent learning-based methods [27, 28], in this paper, we propose an end-to-end image decomposition algorithm via deep learning techniques. A modified fully convolutional network is applied to extract the feature of reconstructed images and suppress the image noise at the same time. The last layer of the model is a fully connected layer to calculate the decomposed images from the extracted features. We demonstrate the effectiveness of our algorithm by the experiment on a clinical dataset. Two conventional algorithms are implemented and compared to the proposed FCN.

## 2. Methods

### 2.1. Fully Convolutional Network

Fully convolutional network (FCN) is one kind of CNN, which is firstly proposed and used for semantic segmentation [29]. The standard CNN generally is composed of a pooling layer and a convolutional layer which are alternately connected. The convolutional layers learn the features of the input. The pooling layers guarantee that the deeper layers can extract higher scale-level features through downsampling. In order to map the feature to the class labels, a fully connected layer will be added to the last output layer, which has fixed dimensions and throws away spatial coordinates. Due to this structural design, the naive CNN requires fixed-sized inputs and produces no-spatial outputs.

The main idea of FCN is transforming the last fully connected layer into a convolution layer with kernels that cover its entire input region. This replacement policy brings about several advantages for FCN. First, the input of the net can be the images of arbitrary sizes, which means that the net can be trained on image patches and then tested on the full-sized images. Second, it can efficiently learn to make dense predictions for per-pixel tasks such as semantic segmentation. Lastly, per-pixel tasks for naive CNN generate a huge amount of redundant convolution computations at adjacent patches. FCN avoids such problems by computing all convolutions in the first layer on the entire input image, leading to significant speedup in the forward-propagation process.

Because of these advantages, FCN is especially suitable for solving the image-based material decomposition problem which can also be regarded as a per-pixel prediction task. In addition, convolution operation to image is interpretable, since it can be seen as a kind of image filtering.

### 2.2. Image Decomposition Model

For image decomposition, we designed an end-to-end decomposition model based on FCN. The proposed model takes reconstructed images as inputs and predicts the basic material coefficients pixel by pixel in the decomposed image, completing image decomposition and noise suppression at one time.

**Figure 1:**
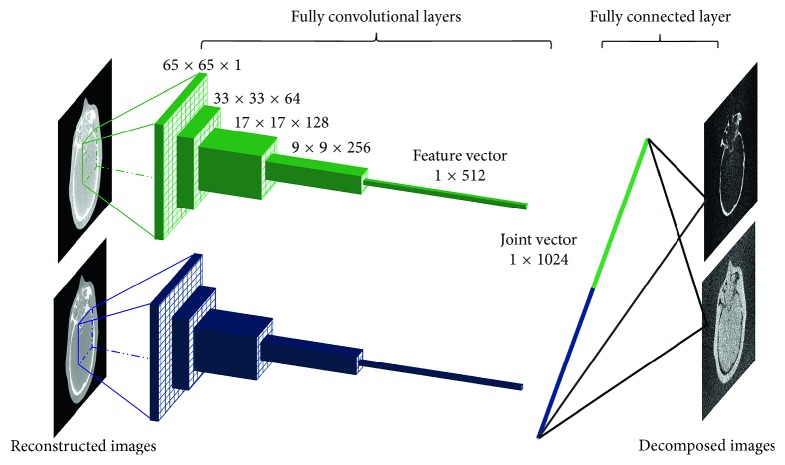
Overall architecture of the proposed network.

An overview of our model is illustrated in Figure 1. It is composed of two types of layers: convolutional and fully connected layers. Since the pooling layer may discard important structural details in feature maps, we omit it from the model to avoid losing the quality of result images. But no downsampling process by the pooling layer will lead to the same size of the feature maps at different layers. We hope the model can still catch the multiscale features of the image at different layers, so the strides of the convolutional layers are set to 2 to finish the downsampling operation. The input of the model is the image patch of 65 × 65 size in reconstructed images. There are two independent fully convolutional nets corresponding to the reconstructed images from low- and high-energy projections. The two nets have the same layer structure and are called the L-FCN and H-FCN in short in this study. They are composed of four convolutional layers. The output of layer *n* can be formulated as follows:

(3) $$\begin{array}{c} C^{n}\left(x_{n}\right)=\mathrm{ReLU}\left(W_{\text{f}}^{n}\;*\;x_{n}+b_{\text{f}}^{n}\right),\;n=1,2,3,4, \end{array}$$

where **x**_*n*_ is the input feature map or images and **W**_f_^*n*^ and **b**_f_^*n*^ represent the convolutional kernel weights and bias parameter, respectively. ∗ is the convolutional operation. ReLU(*x*)=max(0, *x*) is the nonlinear active function of the neuron. The outputs of L-FCN or H-FCN (**C** ^4^(**x**_4_)) are a 512 × 1 vector which represents the feature of the current input patch. The two feature vectors from L-FCN and H-FCN are merged into a joint vector. Then, a fully connected layer is used to calculate the decomposed basic material coefficients from the joint vector, which follows the following equation:

(4) $$\begin{array}{c} X=W_{\text{c}}M+b_{\text{c}}, \end{array}$$

where **X**=(*x*_1_, *x*_2_) is the predicted material coefficients vector, **W**_c_ and **b**_c_ are the unsolved parameter matrixes, and **M** represents the merged vector from L-FCN and H-FCN.

The whole decomposed images can be obtained by traversing all the patches in the input images. The specific information about each layer of the proposed FCN is listed in Table 1.

### 2.3. The Training Detail

The proposed FCN is implemented via the TensorFlow [30] framework on a computer platform containing two Titan X GPUs (a total of 24 GB video memory). The base learning rate of the model is 5 × 10^−3^, which decays by an exponential power of 0.9. There are 1200 training samples in one batch. The mean squared error (MSE) is utilized as the loss function:

(5) $$\begin{array}{c} L\left(W_{\text{c}},b_{\text{c}},W_{\text{f}},b_{\text{f}}\right)=\frac{1}{2}{\left\|X-\hat{X}\right\|}_{2}, \end{array}$$

where $\hat{X}=\left({\hat{x}}_{1},{\hat{x}}_{2}\right)$ is the true value of the decomposed image. We used Adam [31] to optimize the loss function in this study. The entire model contains about 64k unsolved parameters and is trained for 40 epochs in 37 hours. The loss curve for training is plotted in Figure S1 in the Supplementary Materials.

## 3. Experimental Design

### 3.1. Experimental Dataset

The experimental data are acquired from a real clinical dataset which contains 5987 pleural and cranial cavity 512 × 512 images from 12 patients. These raw data are obtained by one single-energy scan. The tissue and bone regions in the images are all manually sketched out. The images from 10 patients were selected to generate training samples, and the images from the rest of the patients were used for testing. All the images are split up into two partitions. Each partition includes regions of bone or tissue only and is used as the ground truth of the decomposed images. In order to generate dual-energy images, we processed the original raw data and simulated the imaging system. The original image is inconvenient to process for its small value. So, firstly, we amplified the value of the raw data to a proper range via a linear transform that follows the following equation:

(6) $$\begin{array}{c} \left\{\begin{array}{c} x_{\text{t}}=\lambda_{\text{t}}{\tilde{x}}_{\text{t}},\\ x_{\text{b}}=\lambda_{\text{b}}{\tilde{x}}_{\text{b}}, \end{array}\right. \end{array}$$

where ${\tilde{x}}_{\text{t}}$ and ${\tilde{x}}_{\text{b}}$ are the pixel values of tissue and bone regions in original images and *x*_t_ and *x*_b_ are the corresponding pixel values in transformed images. The values *λ*_t_ and *λ*_b_ in the experiment are set to 50 and 15, respectively. Here, the different setting of *λ*_t_ and *λ*_b_ is for the purpose of better visual contrast in the transformed images. Secondly, we applied a BM3D [32] algorithm for attenuation of additive white Gaussian noise from the image. Thirdly, we used SpekCalc [33] software to generate 80 kVp and 140 kVp energy spectra, calculated the projection under the simulated scan of dual energy, and obtained the reconstructed images via filtered backprojection (FBP). Lastly, for each patient in the training set, we selected one slice every 10 images. Then, for each image, we extracted 65 × 65 patches with the sliding interval of 5 pixels. The patch size was set to 65 × 65, the same as the input layer of the proposed FCN, getting totally 2,454,300 training patches.

### 3.2. Evaluation Metrics

The proposed FCN is compared with two other algorithms, direct decomposition (matrix inversion) and iterative decomposition [9]. We choose the bias and standard deviation to evaluate the performance of these methods. Bias shows the difference between the measured value and expected value, which can be a measure of the precision of the result. Standard deviation (SD) reflects the degree of dispersion of the result. They are calculated as follows:

(7) $$\begin{array}{c} \text{Bias}=\frac{1}{N}\overset{N}{\underset{i=1}{\sum }}\left|x_{i}-{\hat{x}}_{i}\right|,\\ \text{SD}=\sqrt{\frac{1}{N}\overset{N}{\underset{i=1}{\sum }}{\left(x_{i}-\mu \right)}^{2}}, \end{array}$$

where *x*_*i*_ and ${\hat{x}}_{i}$ are the predicted value and true value at point *i* of the image, respectively, *μ* is the mean value of the material, and *N* is the number of points in ROI.

In order to further investigate the robustness of the proposed FCN, before reconstruction via FBP, photon noise is introduced into the dual-energy projections. There are two major types of noise in X-ray projection images [34]. One type is due to the electrical and roundoff error, which is image independent and can be considered as the Gaussian noise; the other type is due to the statistical fluctuation of the X-ray photons, which is image dependent and can be considered as the Poisson noise. The noise of the first type is small and is omitted in this study. The noise of the second type can be calculated as follows:

(8) $$\begin{array}{c} \left\{\begin{array}{c} {\tilde{p}}_{\text{L}}=\ln I_{\text{L}}-\ln\left(g\left(I_{\text{L}}e^{-p_{\text{L}}}\right)\right),\\ {\tilde{p}}_{\text{H}}=\ln I_{\text{H}}-\ln\left(g\left(I_{\text{H}}e^{-p_{\text{H}}}\right)\right), \end{array}\right. \end{array}$$

where ${\tilde{p}}_{\text{L}}$ and ${\tilde{p}}_{\text{H}}$ are the noise-corrupted low- and high-energy projections, g(*x*) is a random process according to Poisson's distribution with mean *x*, and *I*_L_ and *I*_H_ are the number of photons of low- and high-energy incident X-rays. We set *I*_L_=5 × 10^5^ and *I*_H_=1 × 10^6^ in the experiments, respectively.

## 4. Results

**Figure 2:**
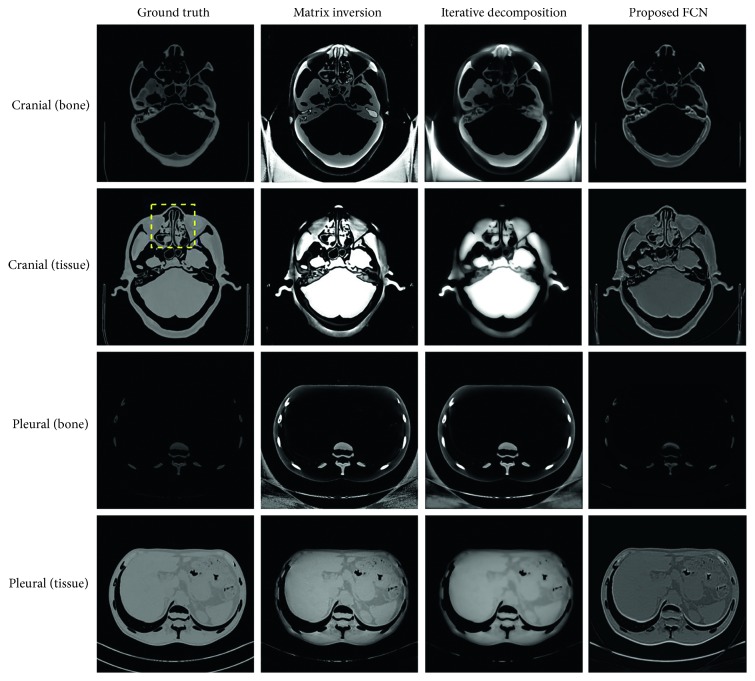
The decomposed images by using three methods.

**Figure 3:**
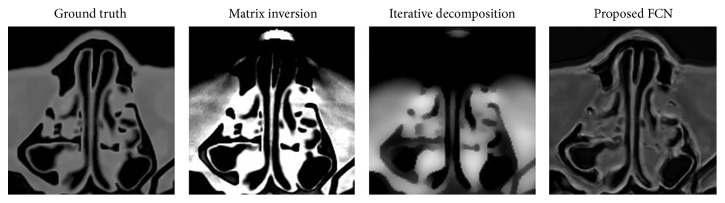
Result comparisons in the zoom-in area which is indicated in Figure 2 with a dashed rectangle.

We test our model on a cranial image and a pleural image which are excluded from the training dataset.  Figure 2 shows the decomposition results by using three algorithms. The first column is the ground truth. Bone and tissue are chosen as the basis materials. Matrix inversion achieves similar results in vision as iterative decomposition. Loss of details and noticeable blocky artifacts are observed for the tissue and bone images from both algorithms.  Figure 3 shows the zoom-in images whose areas are indicated in Figure 2 with a dashed rectangle. The iterative decomposition delivers smooth image due to its smoothness regularization term in loss function. It is noticeable that the proposed FCN suppresses most artifacts while preserving the structural features better than the competing algorithms. But there are not distinct improvements in edge-preserving. We guess this is mainly caused by the convolution kernel in the model. The convolutional operation of image can be seen as a kind of filtering.

For quantitative evaluation, the bias and SD are calculated on the images generated by using different algorithms inside material's ROI and summarized in Table 2. Generally, the estimate of bone is more accurate than that of tissue. The proposed FCN achieves results closest in values to the ground truth, with about 60% smaller bias and 70% lower standard deviation than the competing algorithms, suggesting its better material separation capability.

**Figure 4:**
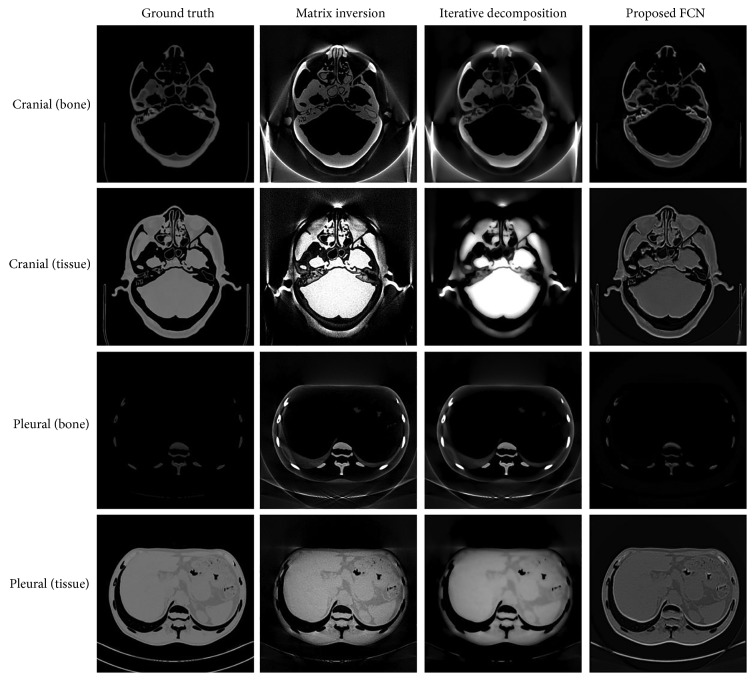
The decomposition results on data with photon noise.

To evaluate the potential improvement by FCN, we investigate the effects of photon noise on the material decomposition algorithms. The reconstructed image is generated from noise-corrupted projections as described in Section 3.2.  Figure 4 presents the decomposition results on same testing images. It can be seen that direct matrix inversion magnifies the noises both in ROI and background. Iterative decomposition also suffers from serious artifacts. This indicates that both algorithms are more sensitive to the noise. The proposed FCN yields the decomposed images that have not much noticeable change in comparison with the results in Figure 2.

**Figure 5:**
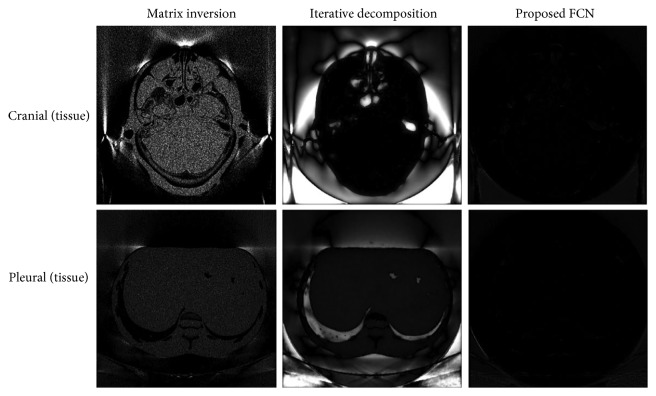
The absolute value of the difference between images in Figures 2 and 4.

Figure 5 illustrates the absolute value of the difference between images in Figures 2 and 4, providing a visual comparison of the performance of noise suppression. For matrix inversion, the noise is statistically independent and evenly distributed in the images because the value of each pixel in decomposed images is calculated by using the corresponding pixel in projections. For iterative decomposition, the noise demonstrates a regional distribution characteristic. The region of tissue and background contain larger amount of noises than bone. In contrast, there are not much obvious differences in the result produced by the proposed FCN. Clearly, it outperforms the other two algorithms, more effectively suppressing image noise while keeping subtle structures.

The quantitative results are listed in Table 3. In the case of photon noise, the bias and SD of the competing algorithms have increased in varying degrees. FCN still demonstrates good agreement to the true value, indicating its advantages on the antinoise capability.

## 5. Discussion

We have designed a cascaded neural network for the material decomposition problem. The reconstructed images are pixel wisely mapped to decomposed images via several convolutional layers and a fully connected layer. The size of the input layer is 65 × 65, based on the hypothesis that the value of the material coefficient depends largely on the local region in reconstructed images. The proposed FCN processes data in an end-to-end way, without any needs of precorrected images or other prior knowledge. The experimental results show its strong performance in capturing the localized structural information and suppressing image noise. The decomposed images generated by matrix inversion and iterative decomposition contain relatively a large amount of artifacts. In the robustness-testing experiment, the noise-corrupted inputs will have a negative impact on the performance of the other competing algorithms, but not much on the FCN. The proposed FCN still achieves excellent results which have low bias and standard deviation. Data augmentation was used in the training process. It brought no boost in performance but costs more training time. We guess the main reason for this issue is that the material decomposition is a regression problem. The value of the label is in a continuous space. Data augmentation assumes that the examples in vicinity share the same class. This hypothesis is usually plausible to the classification problem in which the label is a discrete variable, but unnecessary for the regression problem. The main drawback of our algorithm is the requirement of the specific type of material. Tissue and bone are selected as the basic material in the experiment. The whole model needs to be retrained if one of the materials was changed. So, we hope the proposed algorithm can be used in some applications such as medical diagnosis where the selection of the material is relatively fixed. The amount of training samples is another main factor contributing to the effectiveness of our model. Normally, more data bring better performance of the model. But it may be difficult to collect enough data in some conditions.

## 6. Conclusions and Further Work

In this study, we present a deep learning approach for the image decomposition problem in DECT. According to the preliminary decomposition results, we successfully prove the feasibility of the proposed algorithm which delivers image with 70% smaller bias and 60% lower standard deviation than the competing algorithms. A deep learning paradigm promises to improve the ability of solving the nonlinear problem in DECT.

We think there are two directions of work that are worth further researching. One is to extend our model to make it applicable to the three-materials decomposition problem. The other is the attempt of using the deconvolutional network which will output the whole decomposed images in a forward-propagation calculation rather than pixel wisely prediction.

## Figures and Tables

**Table 1:** Detailed configuration of L-FCN/H-FCN.

Layer name	Kernel size	Stride	Pad	Output size
Input	—	—	—	65 × 65 × 1
Conv1	5 × 5	2	1	33 × 33 × 64
Conv2	5 × 5	2	1	17 × 17 × 128
Conv3	5 × 5	2	1	9 × 9 × 256
Conv4	9 × 9	1	0	1 × 1 × 512

**Table 2:** A list of bias and SD on the images generated by using different algorithms.

Material	Bone (cranial)	Tissue (cranial)	Bone (pleural)	Tissue (pleural)
Matrix inversion	0.410 ± 0.799	0.790 ± 0.930	0.823 ± 1.126	**0.191** **±** **0.348**
Iterative decomposition	0.330 ± 0.621	0.833 ± 1.221	0.763 ± 0.994	0.220 ± 0.417
Proposed FCN	**0.111** **±** **0.280**	**0.283** **±** **0.261**	**0.097** **±** **0.208**	0.322 ± 0.171

**Table 3:** A list of bias and SD on the images in case of photon noise.

Material	Bone (cranial)	Tissue (cranial)	Bone (pleural)	Tissue (pleural)
Matrix inversion	0.425 ± 0.807	0.804 ± 0.983	0.840 ± 1.162	**0.241** **±** **0.390**
Iterative decomposition	0.322 ± 0.608	0.823 ± 1.180	0.773 ± 1.012	0.242 ± 0.423
Proposed FCN	**0.108** **±** **0.284**	**0.283** **±** **0.260**	**0.097** **±** **0.208**	0.290 ± 0.169

## Data Availability

The code and data used in the research can be obtained from https://github.com/XYF-GitHub/ImageDecomposition-DECT.

## Supplementary Materials

Supplementary Materials Figure S1: the proposed model contains about 64k unsolved parameters and is trained for 40 epochs in 37 hours. The training batch size is 1200 reconstructed images from the noise-corrupted low- and high-energy projections. Figures S2 and S3: more testing results to show the superiority of the proposed method. All the testing images are reconstructed from the noise-corrupted low- and high-energy projections.
